# Lonely children and adolescents are less healthy and report less social support: A study on the effect of loneliness on mental health and the moderating role of social support

**DOI:** 10.1186/s12889-025-23247-5

**Published:** 2025-06-26

**Authors:** Raphael Schütz, Franziska Reiss, Irene Moor, Anne Kaman, Ludwig Bilz, HBSC Study Group Germany

**Affiliations:** 1 Department of Health Sciences, Brandenburg University of Technology Cottbus-Senftenberg, Universitätsplatz 1, 01968 Senftenberg, Germany; 2 Department of Child and Adolescent Psychiatry, Psychotherapy, and Psychosomatics, University Medical Center Hamburg-Eppendorf, Hamburg, Germany; 3 Martin Luther University Halle-Wittenberg, Halle (Saale), Germany

**Keywords:** Loneliness, Mental health, Social support, HBSC, Children, Adolescents

## Abstract

**Background:**

Loneliness among children and adolescents has been increasingly recognized as a public health issue, for example, because of its associations with mental health problems. Nevertheless, there is a lack of evidence regarding the links between loneliness and mental health and the potential buffering role of social support. Thus, this study aims to investigate the prevalence of loneliness among children and adolescents in Germany and its associations with mental health. Furthermore, we analyze whether social support is negatively correlated with loneliness and mental health problems and whether it acts as a moderator of the association between loneliness and mental health issues.

**Methods:**

This study analyzed data from the Health Behavior in School-aged Children (HBSC) survey in Germany in 2022, which included 6,475 students aged 11, 13, and 15 years (girls: 50,6%, *M*_*age*_ = 13,4, *SD* = 1,7). Loneliness was measured via the University of California–Los Angeles Scale (UCLA) and a single-item measure. The mental health indicators included subjective health, life satisfaction, and multiple psychosomatic complaints. Social support from family, teachers, and classmates was assessed. Chi-square tests, t tests, logistic regressions, and moderation analyses were conducted.

**Results:**

A total of 17.2% of the students reported high levels of loneliness. Compared with boys, girls and gender-diverse students reported higher rates of loneliness. High levels of loneliness were strongly linked to poorer subjective health (OR = 5.56, *p* <.001), lower life satisfaction (OR = 7.32, *p <*.001), and increased psychosomatic complaints (OR = 7.38, *p* <.001). High social support from family, teachers, and students was associated with reduced loneliness and better mental health outcomes. Teacher support in grades 7 and 9 buffered the effect of loneliness on multiple psychosomatic complaints.

**Conclusion:**

The findings highlight that loneliness is a prevalent phenomenon among children and adolescents and is strongly associated with mental health issues. Greater social support is linked to reduced loneliness and better mental health, so targeted interventions to promote social support in schools and families are needed to address loneliness. Future research should explore longitudinal relationships and further elucidate the mechanisms underlying these associations.

**Supplementary Information:**

The online version contains supplementary material available at 10.1186/s12889-025-23247-5.

## Introduction

Loneliness has become an important public health issue in many countries, especially during the COVID-19 pandemic [1]. Many national and international studies [2–6] have shown increased levels of loneliness in children and adolescents and possible health-related consequences [7]. For example, studies indicate that loneliness is associated with numerous psychosomatic health complaints [3, 8, 9] and psychological burdens such as depression and anxiety [10–12].

Loneliness is an unpleasant feeling due to a quantitative or qualitative lack of social relationships. It is a subjectively perceived and stressful discrepancy between existing and desired relationships [13]. Furthermore, Weiss (1973) distinguished between social loneliness, which involves a lack of social networks, such as within the peer group or at work, and emotional loneliness, described as a lack of intimate attachments, for example, romantic partners and close friendships [14]. For this study, we define loneliness as a distressing feeling resulting from a perceived absence of emotional or social connections.

Loneliness has often been considered a problem of adulthood and older age [15, 16]. However, loneliness also plays an important role in children and adolescents, as they are vulnerable to feelings of loneliness due to the many physical, psychological, and social changes and developmental challenges that occur during this phase of life [17]. Adolescents are still developing their social skills and have not fully formed their identity. They gradually detach themselves from their parents, while peer relationships become more important [18–20], and they must manage sociocultural changes such as going through a school pathway [21]. Coping with these challenges, especially during difficult times such as the pandemic, might affect the mental health of children and adolescents [22]. Accordingly, research shows that unsatisfactory social relationships, such as a lack of friendships, conflicts [20], or a lack of social support, are associated with increased levels of loneliness [23]. Therefore, as one consequence of the pandemic, tracking loneliness and potential buffer factors is proposed as an important research priority to protect public health [7, 24].

However, due to our knowledge, no study has examined the prevalence of loneliness in children and adolescents within this age group and its relationship with mental health or the buffer function of social support between loneliness and mental health. Therefore, the present study addresses this research gap by investigating the prevalence of loneliness in children and adolescents in Germany and examining potential demographic, social, and mental health-related variables. Furthermore, it analyses possible buffering factors such as social support, which is important for addressing loneliness, and derives targeted interventions.

### Prevalence of loneliness in children and adolescents according to sociodemographic factors

The prevalence of loneliness varies depending on the sample, survey country, period, and methods used.

An international school survey investigating loneliness with a single item in 15-year-olds is the Programme for International Student Assessment (PISA) study. In the evaluation of European countries, 13% [25], and in the global assessment, 17.9% [26] of young people felt lonely. Meta-analyses have indicated that the prevalence of loneliness in children and adolescents ranges from 9.2 to 14.4% [27]. In Germany, the prevalence of loneliness in children and adolescents has rarely been investigated in representative studies. In the German COPSY study (COVID-19 and PSYchological Health), students aged 11–17 years were surveyed, whereas 10% of the adolescents reported feeling lonely often, and 24% reported feeling sometimes lonely [28]. However, the COPSY study investigated loneliness with only one item during the pandemic. Thus, there is a lack of representative data with comprehensive loneliness scales for these age groups across Germany.

Taking *sociodemographic factors* into consideration, numerous studies indicate that loneliness is more pronounced in girls than in boys [3, 8, 26], whereas other studies did not find significant gender differences [29] or even slightly greater loneliness in boys [30]. According to age, international studies report greater loneliness in 15-year-old adolescents than in 11-year-old children [3, 9, 31], greater loneliness in 10-year-old children than in 12–16-year-olds [32], or a peak in loneliness among 13-year-olds [12]. The findings are also heterogeneous with respect to socioeconomic status [25, 31] and migration status [25, 26].

In summary, many sociodemographic factors are discussed as possible explanations for higher levels of loneliness. However, the results of these studies remain inconclusive, and further research is needed to clarify the role of specific socio-demographic factors in explaining loneliness. Moreover, the investigation of loneliness with two reliable instruments (a single item and the UCLA Scale) in this age group is lacking. One existing study is from Denmark and uses different response categories in the measurements, which makes it less comparable [8]. Especially in Germany, studies on the prevalence of loneliness in children and adolescents are missing. This will provide valuable insights into a better understanding of loneliness in children and adolescents and possible approaches to prevention.

Additionally, the widespread prevalence across various sociodemographic characteristics emphasizes the importance of examining the potential consequences for public health more closely.

### Loneliness and mental health

Loneliness is often discussed as a potential risk for physical and mental health [2, 33, 34].

According to the definition of the World Health Organization (WHO), mental health is defined as “a state of mental well-being that enables people to cope with the stresses of life, realize their abilities, learn well and work well, and contribute to their community” [35]. Mental health conditions also include psychosocial disabilities, mental health burdens, and other mental states associated with distress and impairments in functioning [35]. On the basis of this definition, the present study focuses on specific positive (e.g., life satisfaction) and negative (e.g., multiple psychosomatic health complaints) aspects of mental health.

Numerous cross-sectional and longitudinal studies have shown links between loneliness and mental health problems such as subjective health [23, 34], depression, and anxiety [2, 11, 36], and multiple psychosomatic health complaints [9, 31]. Furthermore, experimental studies in which loneliness was triggered revealed an increase in symptoms of anxiety and depression, shyness, and a decrease in self-esteem and overall mood [37, 38]. Possible explanations for these links between loneliness and psychological complaints include evolutionary (loneliness causes physical and psychological complaints to avoid group exclusion), physiological (greater activation of the hypothalamic‒pituitary‒adrenal axis, release of more cortisol), psychological (increased vigilance, cognitive bias, lower self-regulation) and behavioral (poorer health behavior) mechanisms [33, 37, 39]. Nevertheless, there is a lack of research on the link between loneliness and mental health, particularly in Germany [40]. Also, internationally, there is still a need for research in this area [8]. The existing studies often focus on psychiatric disorders [36] or physical complaints [8], while other health factors are less considered. Furthermore, most studies examining loneliness and health-related factors focus on adults [15, 33] or older adolescents [23]. Thus, investigating the relationship between loneliness and mental health is crucial for understanding the potential consequences of loneliness in children and adolescents, and for practical applications. For example, to identify possible approaches to reducing loneliness and mitigating the link between loneliness and poorer mental health.

### Loneliness and mental health: the direct effect and moderating role of social support

Social support from family, school, and peers is an important factor in adolescents’ lives, not only for mitigating loneliness [23, 41] but also for their general development and mental health [42–44]. Social support is described as a feeling of appreciation and belonging that contains practical as well as informative and emotional elements (e.g., receiving empathy, care, and help) [45]. For example, school-related support is often described as perceived help, acceptance, interest, and friendliness from classmates and teachers [46], while family support also includes the opportunity to talk about problems [47]. Meta-analysis demonstrated a strong negative correlation between family and friendship support and loneliness [41]. Moreover, support from teachers [23, 25, 26], and classmates [23] is associated with significantly lower levels of loneliness in children and adolescents.

Also, with respect to health, studies indicate that higher levels of peer and teacher support are associated with increased self-esteem, reduced symptoms of anxiety and depression [48], better sleep, and subjective health [23]. Furthermore, greater classmate and parental support is associated with greater life satisfaction [49, 50], and greater peer support is associated with fewer psychosomatic complaints [51].

Two models in particular have gained recognition in the literature as explanations for the significance of social support in mental health [52, 53]. The *main effect model* and the *buffer model*. *The main effect model* assumes that social support provides a general positive effect for a person due to a large social network, which contributes to positive social experience and stable social situations [52, 53]. Thus, social support is directly related to better health, health behavior, well-being, stability, and self-worth [52, 53]. Adapting and expanding this main effect model to address loneliness, social support may fulfill the basic human needs for connection, belonging, and mutual understanding [54]. This support fosters positive emotions, which protects against mental health issues and may help alleviate feelings of loneliness. The *stress buffer model* assumes that social support protects against the effects of stressful events, in particular. Firstly, this means that support can already act as a buffer during the judgment of an event (e.g., the event may be perceived as less stressful) [52]. Secondly, social support can help to act as a buffer by reducing maladaptive emotional, physiological, and behavioral reactions to the event [52, 53, 55].

In adapting to and extending the *buffer model* for loneliness, it could be that the direct effect of loneliness as a stressful feeling on mental health [33, 37] is moderated by social support. Social support may help individuals perceive feelings of loneliness as less stressful and more changeable, thus buffering the possible effects on health. Additionally, social support may mitigate maladaptive reactions to loneliness (e.g., physical stress responses) and buffer possible health consequences. However, due to our knowledge, no other study investigates the possible buffer or main effect model on loneliness in this age group. Furthermore, it could be assumed that the potential moderating function of social support varies by age. For example, a study shows that perceived social support from teachers, classmates, and family declines throughout adolescence [56], while another study suggests that family and friend support remains stable in childhood and youth [57]. Changes in the support received with age could also affect the buffering function. For example, the gradual detachment from the family during adolescence could make school support more important. Thus, teachers in particular are important supportive figures during adolescence [58]. Nevertheless, to our knowledge, no study has investigated possible age differences in the buffering function of social support.

**Fig. 1:**
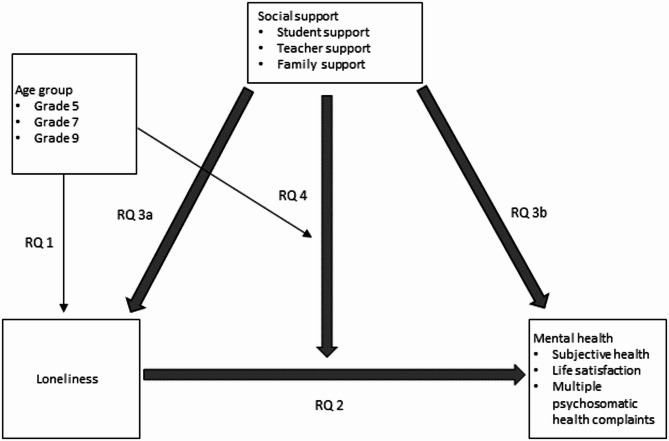
Research question 1–4: the relation between loneliness, social support, and mental health

In summary, adapting the theoretical framework of the main effect and buffering model of social support [52], it can be suggested that social support may have a direct association with loneliness and mental health and plays a moderating role in the relationship between loneliness and its health-related outcomes (see Fig. 1).

However, the current study aims to address several important gaps in the existing body of research. *Firstly*, due to our knowledge, no representative German study has investigated loneliness in this age group. But also internationally, loneliness in children and adolescents is still comparatively rarely studied, and loneliness is rather considered a phenomenon of old age [15, 16]. Moreover, most of the studies use a single item *or* the UCLA scale; the use of both measurements in one study is rare [8]. Existing studies on prevalence differ significantly based on sociodemographic characteristics [3, 8, 30, 32], highlighting the need for more information. *Secondly*, due to our knowledge, no nationwide study in Germany has investigated the connection between loneliness and various mental health aspects in this age group, so a research gap is seen here [40]. Furthermore, the link between loneliness and possible health problems is also being investigated internationally, especially in adulthood and less so in young people [15]. So there is still more research to be done [8]. Some of the existing studies include only younger children [34], older adolescents and adults [23, 26], or are based on a small sample size [34]. *Thirdly*, studies have so far mostly examined single sources of support (e.g., school support) in the context of loneliness [23, 26] or with individual health factors [50]. Studies that consider a comprehensive range of support factors, including both school and family support, are lacking. Furthermore, they either look at the association between support and loneliness [26] or between support and mental health [48], but rarely together. Some studies have assessed social support in older adults [23] and children with specific familial risk factors [45], limiting the applicability to other school children. *Fourthly*, to the best of our knowledge, no study investigates the moderating role of various social support factors between loneliness and mental health indicators, nor possible age differences in this potential buffering role. The present study aims to address these research gaps by exploring the mechanisms underlying loneliness in children and adolescents and examining its potential health consequences. In particular, it investigates both the direct effects of social support on loneliness and mental health, and its potential moderating role. This approach not only contributes to a deeper theoretical understanding but also offers practical implications for the development of targeted prevention and intervention strategies. Based on the outlined research gaps and limitations, the following research questions (RQ) are examined.

- RQ 1. How prevalent is loneliness among children and adolescents in Germany, according to gender, grade level, family affluence, and migration background?
- RQ 2. Is loneliness associated with mental health burdens (lower subjective health, lower life satisfaction, and multiple psychosomatic health complaints)?
- RQ 3. Is perceived social support (from students, teachers, and family) associated with (a) less loneliness and (b) with less mental health burdens?
- RQ 4. If there is an association between loneliness and mental health, does social support moderate this association, and does this moderation vary by age?

## Methods

### Procedure and design

The analyses presented are based on data from the Health Behavior in School-aged Children (HBSC) study in Germany in 2022. The HBSC study is a World Health Organization collaborative cross-national survey and one of the largest child and adolescent health studies. The first German HBSC study took place in 1993/1994. Since then, the survey has been conducted every four years [59]. Each HBSC survey follows a standardized international protocol developed by network members [60]. This protocol includes information regarding the methodology, mandatory, and optional questions of the HBSC survey. In the present German survey, the optional questions on loneliness were incorporated for the first time [60]. Thus, the study aims to collect current data on students’ loneliness, health, social relationships, and sociodemographic factors.

The schools approached for participation in Germany were drawn as a cluster sample from the population of all general education schools. To obtain a representative sample (close to the distribution of the population), the school size (probability proportional to size (PPS) design) and the distribution of students by federal state, school type, grade, and gender were included in the sampling. Permission to conduct the HBSC study in schools was obtained from the relevant state education authorities in each federal state in Germany (except for North Rhine-Westphalia, where schools decide autonomously whether to participate). Approval was granted in close cooperation with the relevant data protection officers. Participation was voluntary and anonymous, with the active consent of school administrators, parents, and students. The adolescents could complete the standardized questionnaire via paper and pencil, online, or offline, with a tablet [59].

### Sample

A total of 174 schools with 7,935 students in grades 5, 7, and 9 (approximately 11, 13, and 15 years old) participated in the 2021/22 survey. The response rates were 8.4% at the school level and 56.8% at the student level. After internationally standardized data cleaning by the HBSC Data Management Centre in Bergen/Norway, quality-neutral omissions in the dataset were corrected (mainly deviations in the age groups, where the variance exceeded +/- 0.5 years). This resulted in a final sample size of *N* = 6,475 (boys: 47.7%, girls: 50.6%, gender-diverse: 1.7%; M_age_ = 13.4, *SD* = 1.7) (see Table 1). Weighting was used to correct the sample regarding federal state, school type, gender, and age group. The final sample is representative of Germany. Further information on the HBSC study in Germany and the methodology can be found in the publication by Winter et al. [59].

**Table 1:** Sample characteristics of the German HBSC survey 2022

Characteristic	*n*	%
**Total**	6,475	100
**Gender**		
Boys	3,074	47.7
Girls	3,258	50.6
Gender-divers	112	1.7
**Grade**		
Grade 5	2,184	33.8
Grade 7	2,201	34
Grade 9	2,080	32.2
**Migration**		
No migration background	4,341	69.2
One-sided migration background	695	11.1
Two-sided migration background	1,240	19.8
**Family Affluence**		
Low	962	15.3
Medium	4,341	68.9
High	996	15.8

*Note* n = sample size; unweighted

### Survey instruments

#### Sociodemographic factors

*Gender* was assessed via three response options: ‘girl’, ‘boy’, or ‘diverse’. The students were capable of indicating their *grade* level directly on the questionnaire. The grade levels included were 5th grade (approximately 11 years), 7th grade (approximately 13 years), and 9th grade (approximately 15 years). *Socioeconomic status* was assessed by the Family Affluence Scale (FAS-III), which covers six items regarding the material assets or activities of children: having one’s own room; the number of cars in the family, holidays abroad; the number of computers at home; how many bedrooms in the home; and whether there is a dishwasher at home [61]. The items were summarized and divided into quintiles, which were grouped into three categories: low (quintile 1– lower bottom 20% of the sample), medium (quintiles 2–4– middle 60% of the sample), and high (quintile 5– top 20% of the sample) [62].

*The migration status* of the participants was operationalized by asking about their country of birth and that of their parents. Young people with one parent who was not born in Germany are categorized as having a one-sided migration background. A two-sided migration background exists if (a) the young person was not born in Germany and at least one parent was not born in Germany or (b) both parents moved to Germany and were not born in Germany.

#### Loneliness

Loneliness was assessed via the 4-item UCLA scale (University of California, Los Angeles) [63]. The UCLA scale is recognized as the ‘gold standard’ loneliness survey [31] and is one of the most commonly used tools for measuring loneliness. The UCLA scale is considered to be reliable and valid [63] and was previously utilized with children and adolescents in the Danish HBSC study [8]. The 20-item version of the UCLA scale has been validated in Germany [64]. For the HBSC study, a shorter 4-item version was translated into German and tested for comprehensibility through a back-translation procedure. In this scale, students rated response options on the basis of how often they experienced certain emotions in the last 12 months (e.g., “I feel left out” or “I am no longer close to anyone”). The response options were as follows: 0 = never, 1 = rarely, 2 = sometimes, 3 = most of the time, and 4 = always. The answers to these four questions were summed to a total score between 0 and 16 points, with the extent of loneliness increasing with increasing score. There are no standardized cutoff values for loneliness [8]. However, it is assumed that a certain level of loneliness is unproblematic [27], while high levels of loneliness are more likely to be associated with health complaints [8, 31]. For this reason, the cutoff value for loneliness, which is based on other studies [3, 4, 31], falls between the middle (‘sometimes’) and the upper two (‘most of the time’ and ‘always’) response categories. This corresponds to a cutoff value of ≥ 9. The aim is to differentiate between occasional and frequent loneliness. This study’s internal consistency of the UCLA scale is very good, with α =.85.

In addition, loneliness was assessed with a single item from the Global Student Health Survey (GSHS) [65]: “During the past 12 months, how often have you felt lonely?” The same response options were used (never, rarely, sometimes, most of the time, always). Previous studies have employed single items to assess loneliness in children and adolescents [5, 26, 31]. Moreover, single items for investigating loneliness have been demonstrated to be highly correlated with multi-item scales [8, 66, 67]. They have also been deemed reliable and valid [66, 68]. On the basis of previous studies [4, 27, 31], a distinction is made between normative and problematic loneliness. Therefore, the cutoff for a single item is defined such that the two upper response categories (mostly and always lonely) are rated as lonely.

#### Mental health factors

##### Multiple psychosomatic health complaints

Data on psychosomatic health complaints were collected via the HBSC Symptom Checklist (HBSC-SCL) [69]. The participants could indicate on a five-point response scale ranging from “about every day” to “rarely or never” how often they had suffered from headaches, stomachache, backache, feeling low, irritability, nervousness, sleeping difficulties, and dizziness in the last six months.

There is no official cut-off point that distinguishes between “normal” and “unbearable” psychosomatic complaints [60, 70]. However, it can be assumed that most people have had one or more of these complaints at least once in the last month [70]. Therefore, the term “multiple psychosomatic health complaints” was used if two or more of these complaints occurred more than once a week. This cut-off value follows the HBSC study protocol guidelines [60] and previous studies [71–74]. This improves the comparability of results, allowing for a distinction between multiple frequent complaints and issues that many people experience occasionally. Thus, psychosomatic complaints are neither pathologized nor dismissed. The scale is considered reliable and valid [75] and, in the current study, has good internal consistency (α = 0.83).

##### Subjective health

The children were asked how they would describe their state of health (poor, fair, good, excellent) [76]. In line with other studies [62, 72], we aimed to differentiate between rather poor and rather good subjective health. Following these previous studies [62, 72] and HBSC reports [73], “poor” and “fair” health were rated as low, and “good” and “excellent” health were rated as high subjective health. The measurement is considered as reliability and valid [76, 77].

##### Life satisfaction

Life satisfaction can be regarded as an aspect of subjective well-being and reflects a person’s overall life situation. To assess this life situation, the students were able to rate how satisfied they were with their lives at the moment using a ladder from 0 to 10 (0 for the worst life possible, 10 for the best life possible) [78]. Following the international HBSC protocol [60] and previous research [62, 72, 79], life satisfaction scores between 0 and 5 were considered low, and life satisfaction scores between 6 and 10 were considered high. The reliability and validity of the life satisfaction item are good [79].

#### Social support

Social support of the adolescents was measured using individual items capturing support from teachers, students, and family.

*Teacher support*: The children and adolescents rated the extent to which they agreed with three statements about their teachers [46] (e.g., “I feel that my teachers accept me as I am” or “I feel a lot of trust in my teachers”). They could choose between the following response options: 0 = strongly agree, 1 = agree, 2 = neither agree nor disagree, 3 = disagree, and 4 = strongly disagree. For better interpretability, the scores were recoded so that higher scores represented higher levels of agreement. In line with the HBSC protocol [60], the three items were combined into a total score (ranging from 0 to 12, with higher values corresponding to greater support). The scale has shown good reliability and validity in previous studies [46, 80] and good internal consistency in the current survey (α = 0.75).

##### Student support

The children and adolescents rated the extent to which they agreed with three statements about their classmates (e.g., “Most of the students in my class are kind and helpful” or “The students in my class enjoy being together”). The response options ranged from 0 = strongly agree to 4 = strongly disagree. The scores were recoded so that higher scores represented higher levels of agreement. Following the HBSC research protocol [60], the three items were combined into a sum score (ranging from 0 to 12, with higher values corresponding to greater support). The scale has been proven valid and reliable [46, 80] and has also shown good internal consistency in a recent study (α = 76).

##### Family support

Family support was assessed via four questions (e.g., “I get the emotional help and support I need from my family”). Students were able to rate their level of disagreement or agreement on a scale from 0 (very strongly disagree) to 6 (very strongly agree) [47]. The individual answers were summarized as scores between 0 (least family support) and 24 (highest family support). The internal consistency is very good, α = 0.92.

### Statistical analysis

To address the first research question, descriptive analyses were performed to investigate the prevalence of loneliness and its correlations with sociodemographic factors via cross-tabulation and chi-square tests. For validation of the bivariate calculations, a binary logistic regression model was calculated with loneliness as the dependent variable and demographic factors as predictors. To address the second research question, chi-squared tests were performed with the binary-coded loneliness scale and the binary-coded mental health factors. To validate the bivariate calculations and also take confounding variables into account, three binary logistic regressions were calculated. In the three regression analyses, loneliness and sociodemographic control variables are the predictors in the first model, and the three mental health factors (subjective health, life satisfaction, multiple psychosomatic health complaints) are the outcomes. To investigate the role of social support, we first analyzed its direct association with loneliness (RQ 3a) and mental health (RQ 3b). To answer question 3a, t-tests and point biserial correlations were first performed. Binary logistic regressions were calculated to verify the bivariate analyses. For this purpose, social support was included in the model as a predictor of loneliness alongside the socioeconomic variables examined in RQ 1 as predictors of loneliness. To answer research question 3b, bivariate analyses (t-tests and point biserial correlations) were also calculated first. Next, binary logistic regressions were performed for each mental health variable as an outcome. Social support was included in the models as an additional predictor alongside loneliness and sociodemographic variables. To answer the fourth research question, the interaction effects (loneliness × social support variables) are included in the logistic model in the third step. In addition, it was examined whether moderations varied depending on age category (grade level). To this end, additional second-order interactions (moderated moderations) were included in the model in a further step (e.g., loneliness x teacher support x grade). Significance is reported via the p-value (significance = *p* <.05), and effect sizes are provided via Cramer’s *V* and Cohen’s *d*. All calculations were performed with SPSS 29.0.

## Results

### Prevalence of loneliness in children and adolescents according to sociodemographic factors (RQ1)

For research question one, a total of *n* = 5,543 children and adolescents responded to the single item, whereas *n* = 5,280 completed the UCLA scale (*M* = 3.76, *SD* = 3.69). Using the cutoff value of ≥ 9 on the UCLA scale, 11.8% of the students were classified as lonely. On the basis of the selected cutoff value for the single item, 17.2% of the children and adolescents reported feeling lonely in the last 12 months (27.4% of the respondents never felt lonely, 28.8% rarely, 26.6% sometimes, 13% most of the time, and 4.2% always). The results of the UCLA scale and single item indicate that both measurements are closely related (χ2 [1] = 1,298.55, *p* <.001, *V* = 0.50). For further analyses, only the results of the more proven and widely used UCLA scale are reported for better clarity. The distribution of loneliness according to sociodemographic characteristics can be found in Table 2.

**Table 2:** Prevalence of loneliness in German students according to sociodemographic factors (*n* = 5.155–5.280)

	Prevalence of loneliness (UCLA scale)			
	Loneliness		Statistical subgroup differences	
	n	%	χ² (df)	*V*
**Gender**				
Boys	186	7.4	165.32(2)***	0.18
Girls	393	14.8		
Gender-divers	42	43.8		
**Grade**				
Grade 5	124	7.7	39.07(2)***	0.09
Grade 7	231	12.6		
Grade 9	267	14.4		
**Family affluence**				
Low	117	12.6	2.29(2)	0.02
Middle	419	12.0		
High	78	10.3		
**Migration**				
No migration	368	11.2	2.48(2)	0.02
One-sided migration	76	12.3		
Two-sided-migration	160	12.8		

Note. Χ² = chi-square, *n* = sample size, *df* = degrees of freedom, *V =* effect size, Cramer’s *V*, *** p <.001

Logistic regression was carried out to validate the bivariate calculations of the subgroup differences and to account for possible confounding factors. In the first step of the regression analysis, loneliness is treated as the criterion variable, with only sociodemographic factors (gender, grade level, family affluence, and migration status) considered predictors (see Table 3, Model 1). The binary logistic regression model was significant (χ2 [8] = 169.36, *p* <.001). Even after all sociodemographic factors were included in the multivariate analyses, the links between loneliness and gender and between loneliness and grade remained significant. For example, girls have a more than twofold increased probability (OR = 2.31, *p* <.001), and gender-diverse students have a ninefold increased probability (OR = 9.01, *p* <.001) of being categorized as lonely compared with boys. In total, the sociodemographic variables explained 6.7% of the variance in loneliness (Nagelkerke *R*^*2*^ = 0.067) (Table 3, Model 1).

**Table 3:** Logistic regression: loneliness by sociodemographic factors and social support (*n* = 4,719)

	Model 1					Model 2				
	B	OR	95%CI		*p*	B	OR	95%CI		*p*
			LL	UL				LL	UL	
**Gender**										
Boys (reference)										
Girls	0.84	2.31	1.90	2.81	< 0.001	0.77	2.20	1.76	2.67	< 0.001
Gender-divers	2.2	9.01	5.74	14.14	< 0.001	1.16	3.18	1.89	5.35	< 0.001
**Grade**										
Grade 5 (reference)										
Grade 7	0.46	1.59	1.24	2.03	< 0.001	0.04	1.04	0.80	1.36	> 0.05
Grade 9	0.60	1.82	1.43	2.32	< 0.001	0.12	1.13	0.87	1.47	> 0.05
**Family affluence**										
Low (reference)										
Middle	0.09	1.10	0.86	1.40	> 0.05	0.16	1.17	0.90	1.52	> 0.05
High	− 0.01	0.99	0.71	1.38	> 0.05	0.17	1.19	0.83	1.70	> 0.05
**Migration**										
No migration (reference)										
One-sided migration	0.19	1.21	0.92	1.59	> 0.05	− 0.08	0.93	0.69	1.25	> 0.05
Two-sided migration	0.15	1.16	0.93	1.44	> 0.05	− 0.12	0.89	0.70	1.3	> 0.05
**Student support**						− 0.22	0.81	0.77	0.84	< 0.001
**Teacher support**						− 0.04	0.96	0.93	0.99	< 0.05
**Family support**						− 0.11	0.90	0.89	0.91	< 0.001
**Model fit**	*R*^*2*^ = 0.067; χ2 (8) = 169.36, *p* <.001					*R*^*2*^ = 0.25; χ2 (11) = 677.13, *p* <.001				

*Note*. B = regression coefficient, CI = confidence interval, OR = odds ratio, *p* = significance, *R*^*2*^ Nagelkerke *R*^*2*^, χ2 = Chi-square. Model 1: Loneliness is the outcome, and sociodemographic factors are predictors (RQ1). Model 2: Social Support variables are added as additional predictors of loneliness (see RQ 3a)

### The association between loneliness and mental health (RQ 2)

For the second research question, bivariate analyses were conducted to examine the associations between loneliness and mental health impairment. A total of 83.8% of children and adolescents state that they are in good or excellent *subjective health*, whereas 16.2% rate their health as fair or poor. Among lonely children and adolescents, only 53.3% reported good or excellent health, while 46.7% reported poor health. Among those who were not lonely, only 12.4% reported poor health, whereas 87.6% reported good health. These group differences are statistically significant (χ2 [1] = 464.81, *p* <.001, *V* = 0.299).

In sum, 86.3% of the respondents reported high *life satisfaction*, and 13.7% reported low life satisfaction. Among the students who felt lonely, only 51.8% reported high life satisfaction, whereas 48.2% reported low satisfaction. In contrast, only 9.7% of nonlonely students rated their health as low, and 90.3% rated it as high. This link between loneliness and life satisfaction is highly significant (χ2 [1] = 654.33, *p* <.001, *V* = 0.355).

A total of 41.7% of students reported suffering from two or more *psychosomatic health complaints* more than once a week, while 83.8% of lonely students suffered from such complaints. In contrast, only 36.9% of students who did not feel lonely stated multiple psychosomatic health complaints. These differences in the multiple psychosomatic complaints are highly significant (χ2 [1] = 488.68, *p* <.001, *V* = 0.307).

To validate the bivariate analyses, binary logistic regressions were calculated for each mental health factor, with loneliness and sociodemographic variables (gender, grade, family affluence and migration) as predictors and the mental health variables (subjective health, life satisfaction and multiple psychosomatic complaints) as binary coded outcomes (see Tables 4, 5 and 6, Modell 1).

The binary logistic regression with *subjective health* as an outcome and loneliness and sociodemographic factors as predictors is statistically significant (χ2 [9] = 470.05, *p* <.001). These predictor variables explain 16% of the variance in poor subjective health (Nagelkerke *R*^*2*^ = 0.16). The probability of reporting poor health is more than five times greater among lonely students than among nonlonely students (OR = 5.56, *p* <.001) (Table 4, Model 1).

**Table 4:** Logistic regression: poor subjective health by sociodemographic factors, loneliness, social support, and interaction effects (*n* = 4,699)

	Model 1				Model 2				Model 3			
	B	OR	95%CI	*p*	B	OR	95%CI	*p*	B	OR	95%CI	*p*
			LL–UL				LL–UL				LL–UL	
**Gender**												
Boys (reference)												
Girls	0.42	1.52	1.28–1.80	< 0.001	0.42	1.53	1.3–1.82	< 0.001	0.43	1.54	1.3–1.83	< 0.001
Gender-divers	1.13	3.09	1.88–5.07	< 0.001	0.60	1.83	1.09–3.05	< 0.05	0.62	1.86	1.1–3.12	< 0.05
**Grade**												
Grade 5 (ref.)												
Grade 7	0.20	1.22	0.98–1.52	> 0.05	− 0.68	0.94	0.74–1.18	> 0.05	− 0.07	0.93	0.74–1.17	> 0.05
Grade 9	0.57	1.77	1.44–2.20	< 0.001	0.25	1.29	1.03–1.61	< 0.05	0.25	1.28	1.03–1.6	< 0.05
**Family affluence**												
Low (ref.)												
Middle	− 0.46	0.63	0.52–0.77	< 0.001	− 0.46	0.63	0.52–0.78	< 0.001	− 0.46	0.63	0.51–0.78	< 0.001
High	-1.07	0.34	0.25–0.47	< 0.001	-1.07	0.34	0.25–0.48	< 0.001	-1.07	0.34	0.25–0.48	< 0.001
**Migration**												
No migration (ref.)												
One-sided migration	− 0.09	0.92	0.71–1.19	> 0.05	− 0.23	0.79	0.60–1.04	> 0.05	− 0.23	0.80	0.61–1.04	> 0.05
Two-sided migration	− 0.30	0.74	0.60–0.91	< 0.05	− 0.45	0.64	0.52–0.79	< 0.001	− 0.44	0.64	0.52–0.80	< 0.001
**Loneliness**												
Not lonely (ref.)												
Lonely	1.72	5.56	4.57–6.77	< 0.001	1.18	3.25	2.62–4.03	< 0.001	0.98	2.65	1.31–5.40	< 0.01
**Student support**					− 0.07	0.93	0.90–0.97	< 0.001	− 0.07	0.94	0.90–0.98	< 0.01
**Teacher support**					− 0.10	0.90	0.87–0.94	< 0.001	− 0.11	0.90	0.86–0.93	< 0.001
**Family support**					− 0.06	0.94	0.93–0.95	< 0.001	− 0.06	0.94	0.92–0.95	< 0.001
**Loneliness x Student support**									− 0.03	0.98	0.90–1.06	> 0.05
**Loneliness x Teacher support**									0.05	1.05	0.97–1.30	> 0.05
**Loneliness x Family support**									0.005	1.0	0.97–1.04	> 0.05
**Model fit**	*R*^*2*^ = 0.16; χ2 (9) = 470.05, *p* <.001				*R*^*2*^ = 0.23; χ2 (12) = 683.04, *p* <.001				*R*^*2*^ = 0.23; χ2 (15) = 684.86, *p* <.001			

*Note*. B = regression coefficient, CI = confidence interval, OR = odds ratio, *p* = significance, *R*^*2*^ = Nagelkerke *R*^*2*^, χ2 = chi-square. Model 1 shows sociodemographic variables and loneliness as predictors and poor subjective health as an outcome (RQ 2). Model 2 includes social support variables as additional predictors of poor subjective health (RQ 3b). Model 3 examines how social support moderates the association between loneliness and poor subjective health (RQ 4)

Table 5 shows that the model with low *life satisfaction* as a criterion and sociodemographic factors and loneliness as predictors is statistically significant (χ2 [9] = 622.95, *p* <.001) and explains 22% of the variance in low life satisfaction (Nagelkerke *R*^*2*^ = 0.22). The probability of low life satisfaction is more than seven times greater for lonely people than for nonlonely people (OR = 7.32, *p* <.001) (Table 5, Model 1).

**Table 5:** Logistic regression: low life satisfaction by sociodemographic factors, loneliness, social support, and interaction effects (*n* = 4,661)

	Model 1				Model 2				Model 3			
	B	OR	95%CI	*p*	B	OR	95%CI	*p*	B	OR	95%CI	*p*
			LL–UL				LL–UL				LL–UL	
**Gender**												
Boys (reference)												
Girls	0.67	1.96	1.61–2.38	< 0.001	0.66	1.94	1.58–2.37	< 0.001	0.67	1.96	1.60–2.41	< 0.001
Gender-divers	1.60	4.93	2.98–8.17	< 0.001	0.96	2.62	1.54–4.45	< 0.001	0.96	2.60	1.53–4.43	< 0.001
**Grade**												
Grade 5 (ref.)												
Grade 7	0.58	1.79	1.39–2.30	< 0.001	0.30	1.35	1.03–1.77	< 0.05	0.30	1.35	1.03–1.76	< 0.05
Grade 9	0.73	2.07	1.61–2.65	< 0.001	0.40	1.49	1.14–1.93	< 0.01	0.39	1.47	1.13–1.92	< 0.01
**Family affluence**												
Low (ref.)												
Middle	− 0.44	0.65	0.52–0.80	< 0.001	− 0.40	0.67	0.53–0.85	< 0.001	− 0.40	0.67	0.53–0.84	< 0.001
High	-1.13	0.32	0.23–0.46	< 0.001	-1.08	0.34	0.23–0.50	< 0.001	-1.07	0.34	0.23–0.50	< 0.001
**Migration**												
No migration (ref.)												
One-sided migration	0.27	1.31	0.99–1.74	> 0.05	0.09	1.10	0.81–1.47	> 0.05	0.09	1.10	0.81–1.50	> 0.05
Two-sided migration	0.46	1.60	1.29–1.95	< 0.001	0.35	1.43	1.14–1.78	< 0.01	0.36	1.44	1.15–1.79	0.001
**Loneliness**												
Not lonely (ref.)												
Lonely	1.99	7.32	5.96–8.98	< 0.001	1.33	3.78	3.0–4.75	< 0.001	1.23	3.40	1.56–7.45	< 0.01
**Student support**					− 0.10	0.90	0.87–0.94	< 0.001	− 0.08	0.92	0.88–0.97	< 0.001
**Teacher support**					− 0.05	0.95	0.92–0.98	< 0.01	− 0.06	0.94	0.90–0.98	< 0.01
**Family support**					− 0.10	0.90	0.89–0.92	< 0.001	− 0.11	0.90	0.89–0.91	< 0.001
**Loneliness x Student support**									− 0.06	0.94	0.86–1.03	> 0.05
**Loneliness x Teacher support**									0.04	1.04	0.96–1.13	> 0.05
**Loneliness x Family support**									0.02	1.02	0.99–1.05	> 0.05
**Model fit**	*R*^*2*^ = 0.22; χ2 (9) = 622.95, *p* <.001				*R*^*2*^ = 0.32; χ2 (12) = 926,91, *p* <.001				*R*^*2*^ = 0.32; χ2 (12) = 930,48, *p* <.001			

*Note*. B = regression coefficient, CI = confidence interval, OR = odds ratio, *p* = significance, *R*^*2*^ = Nagelkerke *R*^*2*^, χ2 = chi-square. Model 1 shows sociodemographic variables and loneliness as predictors and low life satisfaction as an outcome (RQ 2). Model 2 includes social support variables as additional predictors of low life satisfaction (RQ 3b). Model 3 examines how social support moderates the association between loneliness and low life satisfaction (RQ 4)

The first model, shown in Table 6, with *multiple psychosomatic health complaints* as a criterion and sociodemographic factors and loneliness as predictors, is statistically significant (χ2 [9] = 800.06, *p* <.001). These predictor variables explained 21% of the variance in the multiple psychosomatic health complaints (Nagelkerke *R*^*2*^ = 0.21). The probability of suffering from multiple psychosomatic complaints is more than seven times greater for lonely people than for nonlonely people (OR = 7.38, *p* <.001) (Table 6, Model 1).

**Table 6:** Logistic regression: multiple psychosomatic health complaints by sociodemographic factors, loneliness, social support, and interaction effects (*n* = 4,646)

	Model 1				Model 2				Model 3			
	B	OR	95%CI	*p*	B	OR	95%CI	*p*	B	OR	95%CI	*p*
			LL–UL				LL–UL					
**Gender**												
Boys (reference)												
Girls	0.91	2.50	2.19–2.83	< 0.001	0.99	2.69	2.35–3.08	< 0.001	0.99	2.70	2.34–3.07	< 0.001
Gender-divers	2.02	7.55	4.05–14.08	< 0.001	1.51	4.52	2.36–8.65	< 0.001	1.50	4.50	2.34–8.61	< 0.001
**Grade**												
Grade 5 (ref.)												
Grade 7	0.49	1.63	1.38–1.92	< 0.001	0.23	1.26	1.06–1.50	< 0.01	0.23	1.26	1.06–1.50	< 0.01
Grade 9	0.77	2.15	1.83–2.53	< 0.001	0.40	1.49	1.26–1.77	< 0.001	0.40	1.50	1.26–1.78	< 0.001
**Family affluence**												
Low (ref.)												
Middle	0.15	1.16	0.98–1.38	> 0.05	0.18	1.20	1.03–1.43	< 0.05	0.18	1.20	1.0–1.44	< 0.05
High	0.13	1.14	0.91–1.43	> 0.05	0.20	1.22	0.96–1.54	> 0.05	0.20	1.22	0.96–1.55	> 0.05
**Migration**												
No migration (ref.)												
One-sided migration	0.16	1.18	0.96–1.44	> 0.05	0.06	1.07	0.86–1.31	> 0.05	0.06	1.06	0.86–1.31	> 0.05
Two-sided migration	0.16	1.17	1.00–1.37	< 0.05	0.09	1.10	0.93–1.29	> 0.05	0.09	1.09	0.93–1.29	> 0.05
**Loneliness**												
Not lonely (ref.)												
Lonely	2.0	7.38	5.81–9.38	< 0.001	1.53	4.63	3.59–5.97	< 0.001	1.71	5.51	1.97–15.38	0.001
**Student support**					− 0.06	0.94	0.91–0.97	< 0.001	− 0.06	0.94	0.91–0.97	< 0.001
**Teacher support**					− 0.14	0.87	0.84–0.89	< 0.001	− 0.14	0.87	0.85–0.90	< 0.001
**Family support**					− 0.07	0.94	0.93–0.95	< 0.001	− 0.07	0.94	0.93–0.95	< 0.001
**Loneliness x Student support**									0.02	1.02	0.92–1.13	> 0.05
**Loneliness x Teacher support**									− 0.04	0.96	0.88–1.06	> 0.05
**Loneliness x Family support**									− 0.00	1.0	0.96–1.04	> 0.05
**Model fit**	*R*^*2*^ = 0.21; χ2 (9) = 800.06, *p* <.001				*R*^*2*^ = 0.30; χ2 (12) = 1,163.19, *p* <.001				*R*^*2*^ = 0.30; χ2 (12) = 1,163.82, *p* <.001			

*Note*. B = regression coefficient, CI = confidence interval, OR = odds ratio, *p* = significance, *R*^*2*^ = Nagelkerke *R*^*2*^, χ2 = chi-square. Model 1 shows sociodemographic variables and loneliness as predictors and multiple psychosomatic health complaints as an outcome (RQ 2). Model 2 includes social support variables as additional predictors of multiple psychosomatic health complaints (RQ 3b). Model 3 examines how social support moderates the association between loneliness and multiple psychosomatic health complaints (RQ 4)

### The association between social support and loneliness, and mental health (RQ3)

#### Social support and loneliness (RQ 3a)

Overall, the children and adolescents reported high levels of *student support* on a scale of 0–12 (*M* = 8.73, *SD* = 2.41). With respect to research question three, student support is significantly lower among those who feel lonely (*M* = 6.93, *SD* = 2.80) than among nonlonely children and adolescents (*M* = 8.95, *SD* = 2.26) (*t* (714.4) = −17.1, *p* <.001, *d* = − 0.87). Furthermore, point-biserial calculations show that support from students is significantly negatively associated with loneliness (*r* = −.270, *p* <.001). With regard to *teachers’ support*, the students reported a medium level of support (*M* = 7.81, *SD* = 2.72). *Teacher support* is significantly lower for lonely students (*M* = 6.5, *SD* = 3.10) than for nonlonely students (*M* = 8.0, *SD* = 2.60) (*t* (729.6) = −11.50, *p* <.001, *d* = − 0.57). Correlation analysis revealed a significant negative correlation between teacher support and loneliness (*r* = −.18, *p* <.001). *Family support* was rated (on a scale of 0–24) as high by the children and adolescents (*M* = 18.45, *SD* = 6.35). For lonely children, family support was lower (*M* = 12.81, *SD* = 7.10) than for nonlonely children (*M* = 19.20, *SD* = 5.83). These are significant mean differences (*t* (720,8) = −21.2, *p* <.001, *d* = − 0.1.1). The association between family support and loneliness is significantly negative (*r* = −.324, *p* <.001).

To validate the bivariate results, a binary logistic regression was performed with loneliness as the outcome and sociodemographic variables and social support as independent variables (Table 3, Model 2).

The binary logistic regression model was significant (χ2 [11] = 677.13, *p* <.001) and explained 25.5% of the variance in loneliness (Nagelkerke *R*^*2*^ = 0.255). With respect to social support, the probability of being categorized as lonely decreases with increasing levels of student (OR = 0.81, *p* <.001), teacher (OR = 0.96, *p* <.05), and family support (OR = 0.90, *p* <.001) (Table 3, Model 2).

#### Social support and mental health (RQ 3b)

Children and adolescents with poor *subjective health* reported lower levels of student support (*M* = 7.64, *SD* = 2.82 vs. *M* = 8.95, *SD* = 2.30; *t* (1174,7) = −13.36, *p* <.001, *d* = − 0.55), teacher support (M = 6.63, *SD* = 3.06 vs. *M* = 8.04, *SD* = 2.58; *t* (1205.43) = −13.24, *p* <.001, *d* = − 0.53), and family support (*M* = 14.51, *SD* = 7.2 vs. *M* = 19.23, *SD* = 5.84; *t* (1127.35) = −18.39, *p* <.001, *d* = − 0.78) compared with children with good subjective health.

High levels of student support (*r* = −.200, *p* <.001), teacher support (*r* = −.193, *p* <.001), and family support (*r* = −.276, *p* <.001) are in turn negatively related to poor subjective health.

Additionally, among children and adolescents with low *life satisfaction*, student support (*M* = 7.25, *SD* = 2.75 vs. *M* = 8.98, *SD* = 2.24; *t* (933.7) = −16.60, *p* <.001, *d* = − 0.75), teacher support (*M* = 6.46, *SD* = 2.98 vs. *M* = 8.04, *SD* = 2.59; *t* (988.52) = −14.09, *p* <.001, *d* = − 0.60), and family support (*M* = 12.73, *SD* = 7.16 vs. *M* = 19.41, *SD* = 5.65; *t* (903.73) = −24.32, *p* <.001, *d* = -1.14) were lower than those of children with high life satisfaction.

High student support (*r* = −.247, *p* <.001), teacher support (*r* = −.203, *p* <.001), and family support (*r* = −.366, *p* <.001) are negatively related to low life satisfaction.

Students with *multiple psychosomatic complaints* also reported lower levels of student support (*M* = 8.05, *SD* = 2.60 vs. *M* = 9.21, *SD* = 2.13; *t* (4425,71) = −17.75, *p* <.001, *d* = − 0.49), teacher support (*M* = 6.94, *SD* = 2.87 vs. *M* = 8.42, *SD* = 2.42; *t* (4557.81) = −20.32, *p* <.001, *d* = −0.56), and family support (*M* = 16.14, *SD* = 6.97 vs. *M* = 20.06, *SD* = 5.32; *t* (4018.40) = −22.35, *p* <.001, *d* = − 0.65) than did students without multiple psychosomatic complaints. High social support from students (*r* = −.237, *p* <.001), teachers (*r* = −.268, *p* <.001), and family (*r* = −.304, *p* <.001) is again negatively associated with multiple psychosomatic health complaints.

To test whether the social support variables were also associated with mental health in multivariate analysis, we added the social support variables to the logistic regression calculations with sociodemographic variables, loneliness, and social support as predictors (Tables 4, 5 and 6, Model 2) and mental health factors as outcomes.

Regarding *subjective health*, the model is statistically significant (χ2 [12] = 683.03, *p* <.001). It shows that 23% of the variance in subjective health is explained by the predictor variables (Nagelkerke *R*^*2*^ = 0.23). The probability of reporting poor subjective health decreases with increasing social support from students (OR = 0.93, *p* <.001), teachers (OR = 0.90, *p* <.001) and family (OR = 0.94, *p* <.001) (Table 4, Model 2).

Also, the model with *life satisfaction* as outcome is statistically significant (χ2 [12] = 926.90, *p* <.001). With social support variables added it explains 32% of the variance in life satisfaction (Nagelkerke *R*^*2*^ = 0.32). The probability of reporting low life satisfaction decreases with increasing social support from students (OR = 0.90, *p* <.001), teachers (OR = 0.95, *p* <.001) and family (OR = 0.90, *p* <.001) (Table 5, Model 2).

The second model shown in Table 6 is also statistically significant (χ2 [12] = 1163.19, *p* <.001) and explains 30% of the variance in *multiple health complaints* (Nagelkerke *R*^*2*^ = 0.30). Furthermore, the probability of reporting multiple health complaints decreases with increasing student (OR = 0.94, *p* <.001), teacher (OR = 0.87, *p* <.001) and family support (OR = 0.94, *p* <.001).

### Loneliness and mental health: the moderating role of social support (RQ4)

To investigate research question four, whether the relationship between loneliness and mental health is moderated by social support, we calculated regression models with the mental health variables as criteria and loneliness, social support, and sociodemographic variables as predictors. Furthermore, we added two-way interaction effects into Model 3 to investigate the moderating role of social support (loneliness × student support, loneliness × teacher support, loneliness × family support) (see Tables 4, 5 and 6, Model 3).

The relationship between loneliness and *subjective health* is not moderated by student support (OR = 0.98, *p* >.05), teacher support (OR = 1.05, *p* >.05), or family support (OR = 1.0, *p* >.05). The logistic regression with interaction effects for *life satisfaction* also revealed that the link between loneliness and low life satisfaction was not moderated by social support from students (OR = 0.94, *p* >.05), teachers (OR = 1.04, *p* >.05), or family (OR = 1.02, *p* >.05). In terms of *multiple health complaints*, the link between loneliness and multiple psychosomatic complaints is not moderated by students (OR = 1.02, *p* >.05), teachers (OR = 0.96, *p* >.05), or family support (OR = 1.0, *p* >.05). For none of the three aspects of mental health is there more variance explained by the interaction effects than in the models that only analyzed the direct effects of sociodemographic variables, loneliness, and social support.

Next, we examined whether the moderating role of perceived social support (student, teacher, family) in the association between loneliness and mental health differed by age category (grade 5 as the reference compared to grades 7 and 9). To do this, we included two additional two-way interactions (social support variables x grade and loneliness x grade) and then the three-way interactions (moderated moderations) (loneliness x support variables x grade) in the logistic regression in a fourth model (Supplement Tables S1-S3).

For the association between loneliness and *subjective health*, no significant moderation of the social support variables was found depending on age category. Specifically, the three- way- interaction between loneliness, peer support, and grade level was not significant for either grade 7 (*B* = 0.21, OR = 1.23, *p* >.05) or grade 9 (*B* = 0.11, OR = 1.11, *p* >.05). Similarly, no significant interactions emerged for teacher support and grade level (grade 7: *B* =–0.15, OR = 0.86, *p* >.05; grade 9: *B* =–0.14, OR = 0.87, *p* >.05). For family support, no moderated moderation was observed for grade 7 (*B* = 0.09, OR = 1.09, *p* >.05) or grade 9 (*B* = 0.08, OR = 1.08, *p* >.05) (Supplement Table S1).

Furthermore, neither students, teacher, nor family support moderated the relationship between loneliness and *life satisfaction* across grade levels significantly: Loneliness x student support x grade 7 (*B* = − 0.11, OR = 0.90, *p* >.05). Similarly, the three-way interaction between loneliness, student support, and grade 9 also did not yield a significant effect (*B* = − 0.14, OR = 0.87, *p* >.05). Also, the interaction between loneliness, teacher support, and grade 7 was not significant (*B* = − 0.01, OR = 0.99, *p* >.05), nor was the interaction for grade 9 (*B* = − 0.04, OR = 0.96, *p* >.05). In terms of family support, no significant moderation effects were found for the interaction between loneliness, family support, and grade 7 (*B* = 0.02, OR = 1.02, *p* >.05) or for grade 9 (*B* = − 0.02, OR = 0.98, *p* >.05) (Supplement Table S2).

The association between loneliness and *multiple psychosomatic complaints* was also not moderated by peer support, regardless of age category (Grade 7: *B* = − 0.19, OR = 0.83, *p* >.05; Grade 9: *B* = 0.04, OR = 1.04, *p* >.05). In contrast, significant three-way interactions emerged for teacher support. Specifically, the interaction between loneliness, teacher support, and grade 7 was significant (*B* = − 0.28, OR = 0.76, *p* <.05), as was the interaction for grade 9 (*B* = − 0.31, OR = 0.74, *p* <.05). These results suggest that teacher support buffered the negative association between loneliness and multiple psychosomatic complaints more strongly among older students compared to those in grade 5. The interactions between loneliness, family support, and grade 7 (*B* = 0.04, OR = 1.05, *p* >.05) as well as grade 9 (*B* = − 0.006, OR = 0.99, *p* >.05) did not reach statistical significance (Supplement Table S3). Overall, these findings highlight that teacher support, but not peer or family support, moderates the relationship between loneliness and psychosomatic complaints, particularly in older adolescents.

## Discussion

In this study, we determined the prevalence of loneliness among children and adolescents using a representative nationwide sample from Germany. We also analyzed the associations with sociodemographic factors, social support, and mental health.

### Prevalence of loneliness in children and adolescents according to sociodemographic factors (RQ 1)

According to the UCLA scale, 11.8%, and based on the single item, 17.2% of the children and adolescents are lonely. The two results of the survey instruments correlate highly significantly with each other.

With these findings, our study provides, due to our knowledge, the first representative data on loneliness based on two valid and representative survey instruments in this age group in Germany. Previous studies either used only a single item [28] or referred to an older sample [81]. The study thus shows how widespread loneliness is among children and adolescents, both according to direct self-assessment (single item) and indirectly based on several questions (UCLA scale).

The lower prevalence of loneliness according to the UCLA scale than according to the single item could be explained by methodological aspects. High negative scores for all four aspects of loneliness on the UCLA scale are less likely than high negative scores for only one loneliness item. A further possible explanation for this could be that the heightened sense of loneliness portrayed in the media during the coronavirus pandemic has made it easier for young people to relate the term to their personal feelings.

Moreover, the study provides new insights into prevalence differences according to sociodemographic aspects. For example, there is little national or international research into loneliness among children and adolescents from gender-diverse backgrounds. Thus, our findings show that loneliness was more pronounced in gender-diverse students (43.8%) and girls (14.8%) than in boys (7.4%). According to the multivariate analyses, girls have a twofold and gender-diverse ninefold higher probability of being classified as lonely. The higher frequency of loneliness in girls corresponds with the findings of many earlier studies [3, 6, 26, 67] and contrasts with other findings [30] that reported slightly greater levels of loneliness in boys. One potential interpretation for the more pronounced loneliness in girls is frequently attributed to a greater willingness to self-identify as lonely and to admit it to themselves, and a questionnaire [8]. An alternative explanation may be gender-specific socialization, which may encourage girls to open up emotionally and attach greater importance to social relationships [9]. A third interpretation could be that girls are also more vulnerable to other mental health phenomena [3, 72, 82], which are, in turn, linked to loneliness [3, 8, 11]. Greater vulnerability to mental health problems in girls is often explained by biological, psychological, and social differences [83, 84]. The results showing that loneliness is even more pronounced among gender-diverse students could be explained, for example, by the fact that it can be challenging for students to come to terms with their own gender identity. They may also be confronted with greater marginalization and discrimination [85]. The experience of victimization or a lack of acceptance from one’s peers can, in turn, also contribute to feelings of loneliness [20].

In terms of grade level, the results show that loneliness is more pronounced in higher grades. Children and adolescents in ninth grade, in particular, show higher levels of loneliness. These findings substantiate earlier regional [5, 67] and international [3, 31] results. One possible explanation for this may be that, as young people get older, family support diminishes [56, 57], they become more independent from their parents [19], and peer groups become more important [20]. If this transition is not successful, or if relationships with peer groups and romantic partners are not considered satisfying, this can lead to loneliness [20, 86].

### The association between loneliness and mental health (RQ 2)

Lonely students were more than five times more likely to report poor health and more than seven times more likely to report low life satisfaction and multiple psychosomatic complaints. Overall, loneliness and the socioeconomic control variables explain between 16% and 22% of the variance in mental health factors. These results confirm previous international findings investigating links between loneliness and mental health [8, 23, 31]. Nevertheless, most studies to date have focused on the links between loneliness and mental health in adults [15] or older adolescents [23, 25]. Others tend to look at somatic [8] or only one aspect of health [23, 31]. Furthermore, there is a lack of studies on this topic in Germany, especially [40]. Our study, therefore, contributes to understanding the significance of loneliness for a broader spectrum of health factors in this age group. It examines how much loneliness increases the likelihood of several health outcomes, thereby highlighting the relevance of addressing loneliness and its possible consequences. Possible explanations for the link between loneliness and poorer physical and mental health are complex. They include biological, psychological, and social explanations. For example, evolutionary biological explanations suggest that the purpose of loneliness is to cause physical and psychological pain so that people become aware of their loneliness and rejoin the community to ensure a greater likelihood of survival [37, 39]. Another possible biological explanation is that loneliness is associated with increased activity of the HPA axis (hypothalamic‒pituitary‒adrenal axis) [33, 39] and a higher concentration of the stress hormone cortisol [39]. The activity of the HPA axis, in turn, influences numerous physiological, mental, and behavioral factors and regulates, for example, the release of cortisol [39]. An increased concentration of cortisol can, in turn, be associated with poorer physical [87] and mental [88, 89] health. A possible psychological explanation is that lonely people have greater feelings of insecurity and hypervigilance. They activate a type of survival mechanism that leads to increased vigilance and anxiety about threats and social situations [33, 37]. This can lead to a cognitive bias that makes lonely people more likely to expect negative social interactions and, therefore, more likely to react with rejection themselves. These rejecting behaviors may trigger rejecting behavior in potential interaction partners and confirm the negative expectations of lonely people [33]. This self-reinforcing vicious cycle of loneliness is associated with feelings of stress, pessimism, and anxiety [33, 37], which in turn can lead to the activation of other neurobiological mechanisms that influence health [33]. Another possible explanation for the importance of loneliness for health is that self-regulation may be more limited in lonely people [33]. However, self-regulation of feelings, thoughts, and behavior is important for achieving personal goals and adapting to social norms [33]. In turn, poor self-regulation is considered a predictor of numerous negative health-related outcomes [90]. A further possible psychological mechanism linking loneliness to poorer health is the weaker health behaviors often exhibited by lonely individuals, which are subsequently associated with health problems [33]. The results of the study on the effect of loneliness on health also highlight the importance of identifying potential mitigating factors. Social support was therefore examined as one such factor.

### Loneliness and mental health: The main effect and moderating role of social support (RQ 3 and RQ 4)

To investigate the role of social support, we first extended the main effects model of social support [52] and analyzed its direct impact on loneliness (RQ 3a) and mental health (RQ 3b). Following this, we expanded the buffering model to examine the moderation effects of social support between the associations of loneliness and mental health (RQ 4).

#### Direct associations between social support and loneliness (RQ 3a)

The bivariate analyses revealed that high social support from classmates, teachers, and family was associated with lower levels of loneliness. Multivariate analyses also showed that the probability of being classified as lonely decreases significantly as social support increases. The logistic regression model examined that social support and sociodemographic factors explained approximately 25% of the variance in loneliness, whereas the variance explained without the social support variables was only 6.7%. These findings extend existing evidence on the relationship between social support and loneliness [23, 26, 41] to the point that the present study investigates the role of different kinds of social support. While previous studies often analyze only school support [23] or family and peer support [41], our study analyzes both. The result shows that both school and family support contribute to explaining loneliness and reducing the risk of being classified as lonely. Possible explanations for this could be derived from an extended *main effects* model adjusted for loneliness. Accordingly, social support could have a direct effect on the likelihood of experiencing loneliness, for example, by increasing feelings of understanding and belonging through satisfying reciprocal social relationships. This fulfills the basic human need for attachment and prevents loneliness [54]. For example, in childhood, quantitative contact with play partners [20] and the relationship with the family can fulfill this need and protect against loneliness [17]. In adolescence, teachers and peers become more important attachment figures and sources of social support [20, 58]. Thus, a high level of support from family, peers, and teachers may satisfy the need for understanding, acceptance, and trusting relationships and be associated with less loneliness.

#### Direct association between social support and mental health (RQ 3b)

The investigation of the extended main effect model showed that students with a high level of social support are less likely to have poor subjective health, low life satisfaction, and multiple psychosomatic complaints. The findings of this study align with those of previous research, indicating a correlation between social support and aspects of mental health [49, 50]. The results of the current study extend existing findings by examining the effects of multiple sources of social support on multiple mental health variables in a large representative sample. Thus, the results show that the models with the added social support variables explained between 22 and 32% of the variance of the mental health aspects (Tables 4, 5 and 6, Model 2), which is between 7 and 10% points more than the model with only loneliness and socioeconomic variables (Tables 4, 5 and 6, Model 1). The study thus contributes to understanding that both school and family support could have a protective effect on various forms of mental health. It therefore provides key insights for the prevention of mental health problems in children and adolescents. The explanation as to why social support could be so important for mental health can be derived from the extended main effect model. Social support increases feelings of security, satisfaction, self-worth, self-care, and well-being [52, 53]. For example, positive health behaviors (e.g., avoiding drugs, being physically active, and looking for medical help) can be promoted. Additionally, previous findings suggest that social support positively affects physiological stress responses [52, 55].

#### The moderating role of social support (RQ 4)

Given the importance of social support for loneliness and mental health, it could be assumed that, according to an adapted *stress buffer model* [52], social support also acts as a buffer between loneliness as a stressful feeling and mental health.

Therefore, we added interaction effects (e.g., loneliness x student support) across all age categories into the logistic regression models. However, the binary logistic model indicated no moderating role of various types of social support between loneliness and mental health variables (see Tables 4, 5 and 6, Model 3). Comparable studies [23] did not investigate such interaction effects. One possible explanation for the absence of a buffering effect from social support is that loneliness has a more direct influence on the development of loneliness itself. Once individuals become lonely, they may struggle to recognize or accept the social support available to them. This could be, for example, due to a higher level of shyness and social anxiety in lonely people [33, 37]. Thus, loneliness may continue to be perceived as stressful and unchangeable. Therefore, social support may not help to buffer maladaptive reactions to loneliness (e.g., further withdrawal, perceiving social contacts as threatening, and developing physical stress reactions). To check whether social support has a moderating effect in certain age groups, we added moderated moderations in a further model (Loneliness x social support x grade). Here, too, social support in the different age groups did not act as a moderator between loneliness and *subjective health* or loneliness and *life satisfaction*. However, it became apparent that teacher support in grades 7 and 9, in comparison to grade 5, acts as a moderator between loneliness and *multiple psychosomatic complaints*. Possible explanations for the results that social support (from teachers) moderates the effect of loneliness on multiple psychosomatic complaints, but not between loneliness and life satisfaction or subjective health, are complex. One explanation is that loneliness is associated with increased vigilance and stress reactions, which in turn are especially related to psychosomatic complaints [33, 39]. According to the stress buffer model, social support can buffer stress reactions and thus their effects on health [52]. For example, by regulating physical stress reactions [55], promoting positive behaviors, and encouraging people to seek help [52, 53]. The particular importance of teacher support in adolescence compared to childhood could be explained by the fact that adolescents increasingly detach themselves from their parents [18, 19], and teachers become especially important supportive figures, even compared with family and friends [58]. Through their support, they could help to integrate adolescents, alleviate the burden on lonely adolescents, and thus reduce the effect on psychosomatic complaints. Another explanation for why teacher support specifically buffers the effect of loneliness on psychosomatic complaints could also be that loneliness is strongly associated with school stress, which in turn predicts psychosomatic complaints [91]. Teachers could therefore not only alleviate the stress caused by loneliness, but also protect students from the stress of school in higher grades and thus from the multiple psychosomatic health consequences of school-related stress. To our knowledge, this study is the first to provide evidence that specific forms of social support buffer the effect of loneliness on multiple psychosomatic complaints. This contributes to identifying starting points for the prevention of loneliness and potential health consequences. For example, teachers should be made aware of the potential protective and buffering role of their supportive behavior.

### Strengths and limitations

One strength of the current study is that it identified loneliness in a large, representative sample using both a direct measure (a single-item question) and a more complex, indirect tool (the UCLA Loneliness Scale). This approach significantly contributes to our understanding of the prevalence of loneliness in this age group and among three different genders. This study also explores the research gap concerning the direct impact of various forms of social support on loneliness and mental health. Furthermore, to our knowledge, this is the first study investigating the buffering function of different kinds of social support between loneliness and multiple mental health aspects. It therefore offers important contributions to the research on loneliness and potential protective factors and provides information on starting points for interventions. Moreover, this study is the first to examine loneliness in children and adolescents via two valid and reliable survey instruments within a representative German sample.

However, a few limitations need to be mentioned. It could be that although the pandemic and protective measures were ending at the time of the survey, loneliness was still more pronounced among children and adolescents, whereas social relationships were limited. A key limitation of this study is its cross-sectional design, which does not permit conclusions about the causality of the relationships discussed. Loneliness can therefore act both as a risk factor for mental health problems [7, 36, 92] and as a consequence [93] of such impairments. Future research should use longitudinal data to analyze which factors are more likely to act as potential causes and consequences of loneliness. Furthermore, we could examine whether social support acts not as a moderator but as a mediator in this relationship. However, since we wanted to examine the main effect and buffer model, and longitudinal data is recommended for mediation analyses [94], this is a research gap for future studies.

## Conclusion and practical implications

The results of the study show that loneliness is a widespread problem among children and adolescents in Germany and is associated with numerous mental health complaints.

These findings not only characterize loneliness as a potential health issue and thus point to the need for prevention and intervention but also provide possible starting points for such measures. The importance of strengthening social relationships and social support can be deduced from the findings that both loneliness and mental health problems are significantly less pronounced in children and adolescents who experience a high level of social support. Schools are particularly well placed to address this issue because they reach many children, and teachers and classmates play important roles in reducing loneliness and mental health problems.

In particular, the direct effect of teacher support in reducing loneliness and promoting mental health, as well as its buffering role in the link between loneliness and multiple psychosomatic complaints, highlights the importance of fostering a supportive school climate through targeted prevention and intervention efforts.

Furthermore, programs aimed at improving social interaction and enhancing emotional and social skills may be effective in reducing loneliness [95]. Our findings also show that high family support is associated with less loneliness and fewer mental health problems, underlining the importance of involving families in prevention efforts by educating parents about the value of emotional support and appreciative communication. In conclusion, this study underscores that loneliness is a pressing public health concern and highlights the critical role of social support in addressing its impact.

## Electronic supplementary material

Below is the link to the electronic supplementary material.

Supplementary Material 1

## Data Availability

The current HBSC data are not freely available, and their use is restricted exclusively to the HBSC Study Network of Germany for the first 3 years after collection. However, the use of the data by third parties is possible on request. Inquiries about the data or evaluation ideas can be directed to the HBSC Study Network Germany (Head and Coordination: Dr. Irene Moor, Martin Luther University Halle-Wittenberg and Prof. Dr. Kevin Dadaczynski). After access is blocked for 3 years, the national and international HBSC data can be requested from the “HBSC Data Management Centre” (Head: Prof. Oddrun Samdal) at the University of Bergen (Norway).

## Declarations

### Permission to reproduce material from other sources

Not applicable: there is no material from other sources.

## Abbreviations

COPSY: COVID-19 and Psychological Health
COVID: 19-Coronavirus disease 2019
*df*: Degrees of freedom
FAS III: Family Affluence Scale
HBSC: Health behavior in school-aged children
HBSC-SCL: Health Behavior in School-aged Children Symptom Checklist
M: Mean
OR: Odds ratio
UCLA: University of California Los Angeles
SD: Standard deviation
SPSS: Statistical Package for Social Sciences
*V*: Effect size, Cramer’s *V*
WHO: World Health Organization
