# Supplementary material for: Lonely children and adolescents are less healthy and report less social support: A study on the effect of loneliness on mental health and the moderating role of social support
Source: BMC Public Health. 2025 Jun 26;25:2172. doi: 10.1186/s12889-025-23247-5 (PMC12199499; doi:10.1186/s12889-025-23247-5)
Supplement: Supplementary file 1 — Supplementary Material 1 [file 12889_2025_23247_MOESM1_ESM.docx]

**Table S 1**

*Logistic regression: Poor subjective health by sociodemographic factors, loneliness, social support, and interaction effects n = 4,699*

|  | **Model 1** | | | | **Model 2** | | | | **Model 3** | | | | **Model 4** | | | |
| --- | --- | --- | --- | --- | --- | --- | --- | --- | --- | --- | --- | --- | --- | --- | --- | --- |
|  | ***B*** | **OR** | **95%CI** | ***p*** | ***B*** | **OR** | **95%CI** | ***p*** | ***B*** | **OR** | **95%CI** | ***p*** | ***B*** | **OR** | **95%CI** | ***p*** |
|  |  |  | **LL–UL** |  |  |  | **LL–UL** |  |  |  | **LL–UL** |  |  |  | **LL–UL** |  |
| **Gender** |  |  |  |  |  |  |  |  |  |  |  |  |  |  |  |  |
| Boys (reference) |  |  |  |  |  |  |  |  |  |  |  |  |  |  |  |  |
| Girls | .42 | 1.52 | 1.28–1.80 | <.001 | .42 | 1.53 | 1.3–1.82 | <.001 | .43 | 1.54 | 1.3–1.83 | <.001 | .42 | 1.52 | 1.27–1.82 | <.001 |
| Gender-divers | 1.13 | 3.09 | 1.88–5.07 | <.001 | .60 | 1.83 | 1.09–3.05 | <.05 | .62 | 1.86 | 1.1–3.12 | <.05 | .56 | 1.75 | 1.04–2.96 | <.05 |
| **Grade** |  |  |  |  |  |  |  |  |  |  |  |  |  |  |  |  |
| Grade 5 (ref.) |  |  |  |  |  |  |  |  |  |  |  |  |  |  |  |  |
| Grade 7 | .20 | 1.22 | .98–1.52 | >.05 | -.68 | .94 | .74–1.18 | >.05 | -.07 | .93 | .74–1.17 | >.05 | 1.55 | 4.70 | 1.47–15.03 | <.01 |
| Grade 9 | .57 | 1.77 | 1.44–2.20 | <.001 | .25 | 1.29 | 1.03–1.61 | <.05 | .25 | 1.28 | 1.03–1.6 | <.05 | 1.26 | 3.54 | 1.15–10.89 | <.05 |
| **Family affluence** |  |  |  |  |  |  |  |  |  |  |  |  |  |  |  |  |
| Low (ref.) |  |  |  |  |  |  |  |  |  |  |  |  |  |  |  |  |
| Middle | -.46 | .63 | .52–.77 | <.001 | -.46 | .63 | .52–.78 | <.001 | -.46 | .63 | .51–.78 | <.001 | -.46 | .63 | .51–.78 | <.001 |
| High | -1.07 | .34 | .25–.47 | <.001 | -1.07 | .34 | .25–.48 | <.001 | -1.07 | .34 | .25–.48 | <.001 | -1.07 | .34 | .25–.48 | <.001 |
| **Migration** |  |  |  |  |  |  |  |  |  |  |  |  |  |  |  |  |
| No migration (ref.) |  |  |  |  |  |  |  |  |  |  |  |  |  |  |  |  |
| One-sided migration | -.09 | .92 | .71–1.19 | >.05 | -.23 | .79 | .60–1.04 | >.05 | -.23 | .80 | .61–1.04 | >.05 | -.23 | .79 | .61–1.04 | >.05 |
| Two-sided migration | -.30 | .74 | .60–.91 | <.05 | -.45 | .64 | .52–.79 | <.001 | -.44 | .64 | .52–.80 | <.001 | -.45 | .64 | .52–.80 | <.001 |
| **Loneliness** |  |  |  |  |  |  |  |  |  |  |  |  |  |  |  |  |
| Not lonely (ref.) |  |  |  |  |  |  |  |  |  |  |  |  |  |  |  |  |
| Lonely | 1.72 | 5.56 | 4.57–6.77 | <.001 | 1.18 | 3.25 | 2.62–4.03 | <.001 | .98 | 2.65 | 1.31–5.40 | <.01 | 1.84 | 6.31 | 1.23–32.54 | <.05 |
| **Student support** |  |  |  |  | -.07 | .93 | .90–.97 | <.001 | -.07 | .94 | .90–.98 | <.01 | .035 | 1.04 | .94–1.14 | >.05 |
| **Teacher support** |  |  |  |  | -.10 | .90 | .87–.94 | <.001 | -.11 | .90 | .86–.93 | <.001 | -.15 | .86 | .80–.93 | <.001 |
| **Family support** |  |  |  |  | -.06 | .94 | .93–.95 | <.001 | -.06 | .94 | .92–.95 | <.001 | -.04 | .96 | .93–.99 | <.05 |
| **Loneliness x Student support** |  |  |  |  |  |  |  |  | -.03 | .98 | .90–1.06 | >.05 | -.14 | .87 | .72–1.04 | >.05 |
| **Loneliness x Teacher support** |  |  |  |  |  |  |  |  | .05 | 1.05 | .97–1.30 | >.05 | .17 | 1.19 | 1.01–1.42 | >.05 |
| **Loneliness x Family support** |  |  |  |  |  |  |  |  | .005 | 1.0 | .97–1.04 | >.05 | -.06 | .94 | .87–1.01 | >.05 |
| **Loneliness x Grade** |  |  |  |  |  |  |  |  |  |  |  |  |  |  |  |  |
| Loneliness x Grade 5 (ref.) |  |  |  |  |  |  |  |  |  |  |  |  |  |  |  |  |
| Loneliness x Grade 7 |  |  |  |  |  |  |  |  |  |  |  |  | -1.58 | .21 | .03–1.56 | >.05 |
| Loneliness x Grade 9 |  |  |  |  |  |  |  |  |  |  |  |  | -.83 | .44 | -.06–3.13 | >.05 |
| **Student support x Grade** |  |  |  |  |  |  |  |  |  |  |  |  |  |  |  |  |
| Student support x Grade 5 (ref.) |  |  |  |  |  |  |  |  |  |  |  |  |  |  |  |  |
| Student support x Grade 7 |  |  |  |  |  |  |  |  |  |  |  |  | -.16 | .85 | .75­–.96 | <.01 |
| Student support x Grade 9 |  |  |  |  |  |  |  |  |  |  |  |  | -.11 | .90 | .80–1.0 | >.05 |
| **Teacher support x Grade** |  |  |  |  |  |  |  |  |  |  |  |  |  |  |  |  |
| Teacher support x Grade 5 (ref.) |  |  |  |  |  |  |  |  |  |  |  |  |  |  |  |  |
| Teacher support x Grade 7 |  |  |  |  |  |  |  |  |  |  |  |  | .02 | 1.02 | .91–1.13 | >.05 |
| Teacher support x Grade 9 |  |  |  |  |  |  |  |  |  |  |  |  | .06 | 1.06 | .96–1.2 | >.05 |
| **Family support x Grade** |  |  |  |  |  |  |  |  |  |  |  |  |  |  |  |  |
| Family support x Grade 5 (ref.) |  |  |  |  |  |  |  |  |  |  |  |  |  |  |  |  |
| Family support x Grade 7 |  |  |  |  |  |  |  |  |  |  |  |  | -.02 | .98 | .93–1.02 | >.05 |
| Family support x Grade 9 |  |  |  |  |  |  |  |  |  |  |  |  | -.03 | .97 | .93–1.01 | >.05 |
| **Loneliness x Student support x Grade** |  |  |  |  |  |  |  |  |  |  |  |  |  |  |  |  |
| Loneliness x Student support x Grade 5 (ref.) |  |  |  |  |  |  |  |  |  |  |  |  |  |  |  |  |
| Loneliness x Student support x Grade 7 |  |  |  |  |  |  |  |  |  |  |  |  | .21 | 1.23 | .97–1.55 | >.05 |
| Loneliness x Student support x Grade 9 |  |  |  |  |  |  |  |  |  |  |  |  | .11 | 1.11 | .90–1.40 | >.05 |
| **Loneliness x Teacher support x Grade** |  |  |  |  |  |  |  |  |  |  |  |  |  |  |  |  |
| Loneliness x Teacher support x Grade 5 (ref.) |  |  |  |  |  |  |  |  |  |  |  |  |  |  |  |  |
| Loneliness x Teacher support x Grade 7 |  |  |  |  |  |  |  |  |  |  |  |  | -.15 | .86 | .70–1.06 | >.05 |
| Loneliness x Teacher support x Grade 9 |  |  |  |  |  |  |  |  |  |  |  |  | -.14 | .87 | .71–1.07 | >.05 |
| **Loneliness x Family support x Grade** |  |  |  |  |  |  |  |  |  |  |  |  |  |  |  |  |
| Loneliness x Family support x Grade 5 (ref.) |  |  |  |  |  |  |  |  |  |  |  |  |  |  |  |  |
| Loneliness x Family support x Grade 7 |  |  |  |  |  |  |  |  |  |  |  |  | .09 | 1.09 | .99–1.12 | >.05 |
| Loneliness x Family support x Garde 9 |  |  |  |  |  |  |  |  |  |  |  |  | .08 | 1.08 | .99–1.18 | >.05 |
| **Model fit** | *R^2^* = .16; χ2 (9) = 470.05, *p* < .001 | | | | *R^2^* = .23; χ2 (12) = 683.04, *p* < .001 | | | | *R^2^* = .23; χ2 (15) = 684.86, *p* < .001 | | | | *R^2^* = .23; χ2 (29) = 702.49, *p* < .001 | | | |

*Note.* *B =* regression coefficient, CI = confidence interval, OR = odds ratio, *p* = significance, *R^2^* = Nagelkerke *R^2^*, χ2 = chi-square. Model 1 shows sociodemographic variables and loneliness as predictors and subjective health as an outcome (RQ 2). Model 2 includes social support variables as additional predictors of subjective health (RQ 3b). In model 3, interaction effects (loneliness x support variables) were included to examine the moderating role of social support on the association between loneliness and subjective health (RQ 4). In Model 4, additional two- and three-way interaction terms were added to test whether the moderation effect of social support on the association between loneliness and subjective health varies by grade level (RQ 4).

**Table S 2**

*Logistic regression: Low life satisfaction by sociodemographic factors, loneliness, social support, and interaction effects n* = *4,661*

|  | **Model 1** | | | | **Model 2** | | | | **Model 3** | | | | **Model 4** | | | | |
| --- | --- | --- | --- | --- | --- | --- | --- | --- | --- | --- | --- | --- | --- | --- | --- | --- | --- |
|  | ***B*** | **OR** | **95%CI** | ***p*** | ***B*** | **OR** | **95%CI** | ***p*** | ***B*** | **OR** | **95%CI** | ***p*** | ***B*** | **OR** | **95%CI** | ***p*** |  |
|  |  |  | **LL–UL** |  |  |  | **LL–UL** |  |  |  | **LL–UL** |  |  |  | **LL–UL** |  |  |
| **Gender** |  |  |  |  |  |  |  |  |  |  |  |  |  |  |  |  |  |
| Boys (reference) |  |  |  |  |  |  |  |  |  |  |  |  |  |  |  |  |  |
| Girls | .67 | 1.96 | 1.61–2.38 | <.001 | .66 | 1.94 | 1.58–2.37 | <.001 | .67 | 1.96 | 1.60–2.41 | <.001 | .67 | 1.96 | 1.60–2.41 | <.001 |  |
| Gender-divers | 1.60 | 4.93 | 2.98–8.17 | <.001 | .96 | 2.62 | 1.54–4.45 | <.001 | .96 | 2.60 | 1.53–4.43 | <.001 | 1.03 | 2.81 | 1.63–4.82 | <.001 |  |
| **Grade** |  |  |  |  |  |  |  |  |  |  |  |  |  |  |  |  |  |
| Grade 5 (ref.) |  |  |  |  |  |  |  |  |  |  |  |  |  |  |  |  |  |
| Grade 7 | .58 | 1.79 | 1.39–2.30 | <.001 | .30 | 1.35 | 1.03–1.77 | <.05 | .30 | 1.35 | 1.03–1.76 | <.05 | -.35 | .70 | .20–2.45 | >.05 |  |
| Grade 9 | .73 | 2.07 | 1.61–2.65 | <.001 | .40 | 1.49 | 1.14–1.93 | <.01 | .39 | 1.47 | 1.13–1.92 | <.01 | -.45 | .64 | .19–2.20 | >.05 |  |
| **Family affluence** |  |  |  |  |  |  |  |  |  |  |  |  |  |  |  |  |  |
| Low (ref.) |  |  |  |  |  |  |  |  |  |  |  |  |  |  |  |  |  |
| Middle | -.44 | .65 | .52–.80 | <.001 | -.40 | .67 | .53–.85 | <.001 | -.40 | .67 | .53–.84 | <.001 | -.39 | .68 | .54–.86 | .001 |  |
| High | -1.13 | .32 | .23–.46 | <.001 | -1.08 | .34 | .23–.50 | <.001 | -1.07 | .34 | .23–.50 | <.001 | -1.06 | .35 | .24–.51 | <.001 |  |
| **Migration** |  |  |  |  |  |  |  |  |  |  |  |  |  |  |  |  |  |
| No migration (ref.) |  |  |  |  |  |  |  |  |  |  |  |  |  |  |  |  |  |
| One-sided migration | .27 | 1.31 | .99–1.74 | >.05 | .09 | 1.10 | .81­–1.47 | >.05 | .09 | 1.10 | .81–1.50 | >.05 | .08 | 1.09 | .80–1.47 | >.05 |  |
| Two-sided migration | .46 | 1.60 | 1.29–1.95 | <.001 | .35 | 1.43 | 1.14–1.78 | <.01 | .36 | 1.44 | 1.15–1.79 | .001 | .35 | 1.42 | 1.14–1.78 | <.01 |  |
| **Loneliness** |  |  |  |  |  |  |  |  |  |  |  |  |  |  |  |  |  |
| Not lonely (ref.) |  |  |  |  |  |  |  |  |  |  |  |  |  |  |  |  |  |
| Lonely | 1.99 | 7.32 | 5.96–8.98 | <.001 | 1.33 | 3.78 | 3.0–4.75 | <.001 | 1.23 | 3.40 | 1.56–7.45 | <.01 | .14 | 1.15 | .21–6.23 | >.05 |  |
| **Student support** |  |  |  |  | -.10 | .90 | .87­–.94 | <.001 | -.08 | .92 | .88–.97 | <.001 | -.11 | .90 | .81–.99 | <.05 |  |
| **Teacher support** |  |  |  |  | -.05 | .95 | .92–.98 | <.01 | -.06 | .94 | .90–.98 | <.01 | -.11 | .90 | .82–.99 | <.05 |  |
| **Family support** |  |  |  |  | -.10 | .90 | .89–.92 | <.001 | -.11 | .90 | .89–.91 | <.001 | -.10 | .90 | .87–.93 | <.001 |  |
| **Loneliness x Student support** |  |  |  |  |  |  |  |  | -.06 | .94 | .86–1.03 | >.05 | .04 | 1.04 | .86–1.27 | >.05 |  |
| **Loneliness x Teacher support** |  |  |  |  |  |  |  |  | .04 | 1.04 | .96–1.13 | >.05 | .05 | 1.05 | .87–1.27 | >.05 |  |
| **Loneliness x Family support** |  |  |  |  |  |  |  |  | .02 | 1.00 | .99–1.05 | >.05 | .02 | 1.02 | .94–1.10 | >.05 |  |
| **Loneliness x Grade** |  |  |  |  |  |  |  |  |  |  |  |  |  |  |  |  |  |
| Loneliness x Grade 5 (ref.) |  |  |  |  |  |  |  |  |  |  |  |  |  |  |  |  |  |
| Loneliness x Grade 7 |  |  |  |  |  |  |  |  |  |  |  |  | 1.31 | 3.70 | .44–31.34 | >.05 |  |
| Loneliness x Grade 9 |  |  |  |  |  |  |  |  |  |  |  |  | 1.50 | 4.47 | .55–35.99 | >.05 |  |
| **Student support x Grade** |  |  |  |  |  |  |  |  |  |  |  |  |  |  |  |  |  |
| Student support x Grade 5 (ref.) |  |  |  |  |  |  |  |  |  |  |  |  |  |  |  |  |  |
| Student support x Grade 7 |  |  |  |  |  |  |  |  |  |  |  |  | .002 | 1.0 | .88–1.15 | <.01 |  |
| Student support x Grade 9 |  |  |  |  |  |  |  |  |  |  |  |  | .06 | 1.06 | .93–1.20 | >.05 |  |
| **Teacher support x Grade** |  |  |  |  |  |  |  |  |  |  |  |  |  |  |  |  |  |
| Teacher support x Grade 5 (ref.) |  |  |  |  |  |  |  |  |  |  |  |  |  |  |  |  |  |
| Teacher support x Grade 7 |  |  |  |  |  |  |  |  |  |  |  |  | .06 | 1.06 | .94–1.20 | >.05 |  |
| Teacher support x Grade 9 |  |  |  |  |  |  |  |  |  |  |  |  | .05 | 1.05 | .94–1.18 | >.05 |  |
| **Family support x Grade** |  |  |  |  |  |  |  |  |  |  |  |  |  |  |  |  |  |
| Family support x Grade 5 (ref.) |  |  |  |  |  |  |  |  |  |  |  |  |  |  |  |  |  |
| Family support x Grade 7 |  |  |  |  |  |  |  |  |  |  |  |  | -.002 | 1.0 | .95–1.04 | >.05 |  |
| Family support x Grade 9 |  |  |  |  |  |  |  |  |  |  |  |  | -.001 | 1.0 | .96–1.04 | >.05 |  |
| **Loneliness x Student support x Grade** |  |  |  |  |  |  |  |  |  |  |  |  |  |  |  |  |  |
| Loneliness x Student support x Grade 5 (ref.) |  |  |  |  |  |  |  |  |  |  |  |  |  |  |  |  |  |
| Loneliness x Student support x Grade 7 |  |  |  |  |  |  |  |  |  |  |  |  | -.11 | .90 | .70–1.16 | >.05 |  |
| Loneliness x Student support x Grade 9 |  |  |  |  |  |  |  |  |  |  |  |  | -.14 | .87 | .68–1.11 | >.05 |  |
| **Loneliness x Teacher support x Grade** |  |  |  |  |  |  |  |  |  |  |  |  |  |  |  |  |  |
| Loneliness x Teacher support x Grade 5 (ref.) |  |  |  |  |  |  |  |  |  |  |  |  |  |  |  |  |  |
| Loneliness x Teacher support x Grade 7 |  |  |  |  |  |  |  |  |  |  |  |  | -.01 | .99 | .79–1.24 | >.05 |  |
| Loneliness x Teacher support x Grade 9 |  |  |  |  |  |  |  |  |  |  |  |  | -.04 | .96 | .77–1.21 | >.05 |  |
| **Loneliness x Family support x Grade** |  |  |  |  |  |  |  |  |  |  |  |  |  |  |  |  |  |
| Loneliness x Family support x Grade 5 (ref.) |  |  |  |  |  |  |  |  |  |  |  |  |  |  |  |  |  |
| Loneliness x Family support x Grade 7 |  |  |  |  |  |  |  |  |  |  |  |  | .02 | 1.02 | .93–1.12 | >.05 |  |
| Loneliness x Family support x Garde 9 |  |  |  |  |  |  |  |  |  |  |  |  | -.02 | .98 | .90–1.80 | >.05 |  |
| **Model fit** | *R^2^* = .22; χ2 (9) = 622.95, *p* < .001 | | | | *R^2^* = .32; χ2 (12) = 926.91, *p* < .001 | | | | *R^2^* = .32; χ2 (12) = 930.48, *p* < .001 | | | | *R^2^* = .33; χ2 (29) = 947.25, *p* < .001 | | | | |

*Note.* *B =* regression coefficient, CI = confidence interval, OR = odds ratio, *p* = significance, *R^2^* = Nagelkerke *R^2^*, χ2 = chi-square. Model 1 shows sociodemographic variables and loneliness as predictors and life satisfaction as an outcome (RQ 2). Model 2 includes social support variables as additional predictors of life satisfaction (RQ 3b). In model 3, interaction effects (loneliness x support variables) were included to examine the moderating role of social support on the association between loneliness and life satisfaction (RQ 4). In Model 4, additional two- and three-way interaction terms were added to test whether the moderation effect of social support on the association between loneliness and life satisfaction varies by grade level (RQ 4).

**Table S 3**

*Logistic regression: Multiple psychosomatic health complaints by sociodemographic factors, loneliness, social support, and interaction effects n = 4,646*

|  | **Model 1** | | | | **Model 2** | | | | **Model 3** | | | | **Model 4** | | | | |
| --- | --- | --- | --- | --- | --- | --- | --- | --- | --- | --- | --- | --- | --- | --- | --- | --- | --- |
|  | ***B*** | **OR** | **95%CI** | ***p*** | ***B*** | **OR** | **95%CI** | ***p*** | ***B*** | **OR** | **95%CI** | ***p*** | ***B*** | **OR** | **95%CI** | ***p*** |  |
|  |  |  | **LL–UL** |  |  |  | **LL–UL** |  |  |  | **LL–UL** |  |  |  | **LL–UL** |  |  |
| **Gender** |  |  |  |  |  |  |  |  |  |  |  |  |  |  |  |  |  |
| Boys (reference) |  |  |  |  |  |  |  |  |  |  |  |  |  |  |  |  |  |
| Girls | .91 | 2.50 | 2.19–2.83 | <.001 | .99 | 2.69 | 2.35–3.08 | <.001 | .99 | 2.70 | 2.34–3.07 | <.001 | .98 | 2.65 | 2.32–3.04 | <.001 |  |
| Gender-divers | 2.02 | 7.55 | 4.05–14.08 | <.001 | 1.51 | 4.52 | 2.36–8.65 | <.001 | 1.50 | 4.50 | 2.34–8.61 | <.001 | 1.51 | 4.53 | 2.36–8.70 | <.001 |  |
| **Grade** |  |  |  |  |  |  |  |  |  |  |  |  |  |  |  |  |  |
| Grade 5 (ref.) |  |  |  |  |  |  |  |  |  |  |  |  |  |  |  |  |  |
| Grade 7 | .49 | 1.63 | 1.38–1.92 | <.001 | .23 | 1.26 | 1.06–1.50 | <.01 | .23 | 1.26 | 1.06–1.50 | <.01 | .71 | 2.03 | .80­–5.11 | >.05 |  |
| Grade 9 | .77 | 2.15 | 1.83–2.53 | <.001 | .40 | 1.49 | 1.26–1.77 | <.001 | .40 | 1.50 | 1.26–1.78 | <.001 | .24 | 1.27 | .51–3.15 | >.05 |  |
| **Family affluence** |  |  |  |  |  |  |  |  |  |  |  |  |  |  |  |  |  |
| Low (ref.) |  |  |  |  |  |  |  |  |  |  |  |  |  |  |  |  |  |
| Middle | .15 | 1.16 | .98–1.38 | >.05 | .18 | 1.20 | 1.03–1.43 | <.05 | .18 | 1.20 | 1.0–1.44 | <.05 | .18 | 1.20 | 1.0–1.44 | <.05 |  |
| High | .13 | 1.14 | .91–1.43 | >.05 | .20 | 1.22 | .96–1.54 | >.05 | .20 | 1.22 | .96–1.55 | >.05 | .21 | 1.24 | .97–1.57 | >.05 |  |
| **Migration** |  |  |  |  |  |  |  |  |  |  |  |  |  |  |  |  |  |
| No migration (ref.) |  |  |  |  |  |  |  |  |  |  |  |  |  |  |  |  |  |
| One-sided migration | .16 | 1.18 | .96–1.44 | >.05 | .06 | 1.07 | .86–1.31 | >.05 | .06 | 1.06 | .86–1.31 | >.05 | .08 | 1.08 | .87–1.33 | >.05 |  |
| Two-sided migration | .16 | 1.17 | 1.00–1.37 | <.05 | .09 | 1.10 | .93–1.29 | >.05 | .09 | 1.09 | .93–1.29 | >.05 | .09 | 1.10 | .93–1.29 | >.05 |  |
| **Loneliness** |  |  |  |  |  |  |  |  |  |  |  |  |  |  |  |  |  |
| Not lonely (ref.) |  |  |  |  |  |  |  |  |  |  |  |  |  |  |  |  |  |
| Lonely | 2.0 | 7.38 | 5.81–9.38 | <.001 | 1.53 | 4.63 | 3.59–5.97 | <.001 | 1.71 | 5.51 | 1.97–15.38 | .001 | .64 | 1.89 | .30–11.82 | >.05 |  |
| **Student support** |  |  |  |  | -.06 | .94 | .91–.97 | <.001 | -.06 | .94 | .91–.97 | <.001 | -.07 | .94 | .88–.99 | <.05 |  |
| **Teacher support** |  |  |  |  | -.14 | .87 | .84–.89 | <.001 | -.14 | .87 | .85–.90 | <.001 | -.15 | .86 | .81–.91 | <.001 |  |
| **Family support** |  |  |  |  | -.07 | .94 | .93–.95 | <.001 | -.07 | .94 | .93–.95 | <.001 | -.05 | .95 | .93–.97 | <.001 |  |
| **Loneliness x Student support** |  |  |  |  |  |  |  |  | .02 | 1.02 | .92–1.13 | >.05 | .04 | 1.04 | .85–1.26 | >.05 |  |
| **Loneliness x Teacher support** |  |  |  |  |  |  |  |  | -.04 | .96 | .88–1.06 | >.05 | .15 | 1.16 | .96–1.41 | >.05 |  |
| **Loneliness x Family support** |  |  |  |  |  |  |  |  | -.00 | 1.0 | .96–1.04 | >.05 | -.02 | .98 | .91–1.06 | >.05 |  |
| **Loneliness x Grade** |  |  |  |  |  |  |  |  |  |  |  |  |  |  |  |  |  |
| Loneliness x Grade 5 (ref.) |  |  |  |  |  |  |  |  |  |  |  |  |  |  |  |  |  |
| Loneliness x Grade 7 |  |  |  |  |  |  |  |  |  |  |  |  | 2.69 | 14.74 | .84–­259.2 | >.05 |  |
| Loneliness x Grade 9 |  |  |  |  |  |  |  |  |  |  |  |  | 1.72 | 5.60 | .43–73.41 | >.05 |  |
| **Student Support x Grade** |  |  |  |  |  |  |  |  |  |  |  |  |  |  |  |  |  |
| Student support x Grade 5 (ref.) |  |  |  |  |  |  |  |  |  |  |  |  |  |  |  |  |  |
| Student support x Grade 7 |  |  |  |  |  |  |  |  |  |  |  |  | -.07 | .93 | .85–1.01 | >.05 |  |
| Student support x Grade 9 |  |  |  |  |  |  |  |  |  |  |  |  | .08 | 1.08 | .99–1.17 | >.05 |  |
| **Teacher support x Grade** |  |  |  |  |  |  |  |  |  |  |  |  |  |  |  |  |  |
| Teacher support x Grade 5 (ref.) |  |  |  |  |  |  |  |  |  |  |  |  |  |  |  |  |  |
| Teacher support x Grade 7 |  |  |  |  |  |  |  |  |  |  |  |  | .07 | 1.07 | .99–1.15 | >.05 |  |
| Teacher support x Grade 9 |  |  |  |  |  |  |  |  |  |  |  |  | -.01 | .99 | .92–1.07 | >.05 |  |
| **Family support x Grade** |  |  |  |  |  |  |  |  |  |  |  |  |  |  |  |  |  |
| Family support x Grade 5 (ref.) |  |  |  |  |  |  |  |  |  |  |  |  |  |  |  |  |  |
| Family support x Grade 7 |  |  |  |  |  |  |  |  |  |  |  |  | -.02 | .98 | .95–1.02 | >.05 |  |
| Family support x Grade 9 |  |  |  |  |  |  |  |  |  |  |  |  | -.02 | .98 | .95–1.01 | >.05 |  |
| **Loneliness x Student support x Grade** |  |  |  |  |  |  |  |  |  |  |  |  |  |  |  |  |  |
| Loneliness x Student support x Grade 5 (ref.) |  |  |  |  |  |  |  |  |  |  |  |  |  |  |  |  |  |
| Loneliness x Student support x Grade 7 |  |  |  |  |  |  |  |  |  |  |  |  | -.19 | .83 | .61–1.11 | >.05 |  |
| Loneliness x Student support x Grade 9 |  |  |  |  |  |  |  |  |  |  |  |  | .04 | 1.04 | .80–1.34 | >.05 |  |
| **Loneliness x Teacher support x Grade** |  |  |  |  |  |  |  |  |  |  |  |  |  |  |  |  |  |
| Loneliness x Teacher support x Grade 5 (ref.) |  |  |  |  |  |  |  |  |  |  |  |  |  |  |  |  |  |
| Loneliness x Teacher support x Grade 7 |  |  |  |  |  |  |  |  |  |  |  |  | -.28 | .76 | .59–.97 | <.05 |  |
| Loneliness x Teacher support x Grade 9 |  |  |  |  |  |  |  |  |  |  |  |  | -.31 | .74 | .57–.95 | <.05 |  |
| **Loneliness x Family support x Grade** |  |  |  |  |  |  |  |  |  |  |  |  |  |  |  |  |  |
| Loneliness x Family support x Grade 5 (ref.) |  |  |  |  |  |  |  |  |  |  |  |  |  |  |  |  |  |
| Loneliness x Family support x Grade 7 |  |  |  |  |  |  |  |  |  |  |  |  | .04 | 1.05 | .94–1.16 | >.05 |  |
| Loneliness x Family support x Garde 9 |  |  |  |  |  |  |  |  |  |  |  |  | -.006 | .99 | .90–1.1 | >.05 |  |
| **Model fit** | *R^2^* = .21; χ2 (9) = 800.06, *p* < .001 | | | | *R^2^* = .30; χ2 (12) = 1,163.19, *p* < .001 | | | | *R^2^* = .30; χ2 (15) = 1,163.82, *p* < .001 | | | | *R^2^* = .31; χ2 (29) = 1,199.25, *p* < .001 | | | | |

*Note.* *B =* regression coefficient, CI = confidence interval, OR = odds ratio, *p* = significance, *R^2^* = Nagelkerke *R^2^*, χ2 = chi-square. Model 1 shows sociodemographic variables and loneliness as predictors and multiple psychosomatic health complaints as an outcome (RQ 2). Model 2 includes social support variables as additional predictors of multiple psychosomatic health complaints (RQ 3b). In model 3, interaction effects (loneliness x support variables) were included to examine the moderating role of social support on the association between loneliness and multiple health complaints (RQ 4). In Model 4, additional two- and three-way interaction terms were added to test whether the moderation effect of social support on the association between loneliness and multiple psychosomatic health complaints varies by grade level (RQ 4).
